# Modulation of cellular signaling pathways in P23H rhodopsin photoreceptors

**DOI:** 10.1016/j.cellsig.2013.12.008

**Published:** 2013-12-27

**Authors:** Olga S. Sizova, Vishal M. Shinde, Austin R. Lenox, Marina S. Gorbatyuk

**Affiliations:** a University of North Texas Health Science Center, North Texas Eye Research Institute, Department of Cell Biology and Anatomy, United States; b University of Alabama at Birmingham, Department of Visual Sciences, United States

**Keywords:** UPR, Calpains, Mitochondria, mTOR, Autophagy, Rapamycin

## Abstract

We previously reported activation of the unfolded protein response (UPR) in P23H rhodopsin (RHO) retinas with autosomal dominant retinitis pigmentosa (ADRP). Knowing that the UPR can trigger Ca^2+^ release from the endoplasmic reticulum and regulate cellular signaling we examined the level of Ca^2+^-regulated proteins. We also looked for changes in the expression of Bcl2 family proteins, autophagy proteins and the mTOR/AKT pathways, as well as for the induction of mitochondria-associated apoptosis in the P23H RHO retina. Our data demonstrated that the elevation of calpain and caspase-12 activity was concomitantly observed with a decrease in the BCL2-XL/BAX ratio and an increase in mTor levels in the P23H-3 RHO retina suggesting a vulnerability of P23H RHO photoreceptors to apoptosis. The translocation of BAX to the mitochondria, as well as the release of cytochrome C and AIF into the cytosol supports this conclusion and indicates the involvement of mitochondria-induced apoptosis in the progression of ADRP. The level of autophagy proteins in general was found to be decreased in the P21–P30 P23H RHO retina. Injections of rapamycin, however, protected the P23H RHO rod photoreceptors from experiencing physiological decline. Despite this fact, the downregulation of mTOR did not alter the level of autophagy proteins. Our results imply that in addition to activation of the UPR during ADRP progression, photoreceptors also experience alterations in major proapoptotic pathways.

## 1. Introduction

Retinitis pigmentosa (RP) is a heterogeneous group of eye disorders that result in irreversible blindness. Visual symptoms include night blindness, followed by photopia and decreasing visual fields, leading to tunnel vision and eventually legal blindness [1,2]. Most frequently autosomal dominant RP (ADRP) is associated with mutations in rhodopsin (RHO), with such mutations accounting for approximately 25% of all ADRP cases [1]. Based on their biochemical and cellular properties, ADRP rhodopsin mutations have been classified into six groups, however most fall into the class I or class II categories [3]. Class I mutations can fold normally, but are not correctly transported to the outer segment. The S334ter RHO protein expressed in transgenic rats represents a Class I mutation. The substitution of proline for histidine at position 23 within the rhodopsin (P23H RHO) gene yields a Class II mutation. These RHO mutants are defective for proper folding. They are retained in the endoplasmic reticulum (ER) and are unable to form a functional chromophore with 11-cis-retinal. They are then transported to the cytoplasm for degradation by the proteasome [4].

In 1990 the P23H RHO mutant was proposed to cause ADRP [5]. Since then, the expression of P23H mutant rhodopsin (RHO) in photoreceptors has been studied in transgenic mice [6-8], rats [9-11] and frogs[12-14]. Loss of photoreceptors and the course of visual decline have been previously described for rats expressing both the S334ter and P23H RHO transgenes (http://www.ucsfeye.net/mlavailRDratmodels.shtml) [15,16]. However, molecular mechanism involved in the loss of vision in Class I and II mutations has not been studied in detail. Previously, we have demonstrated that activation of the unfolded protein response (UPR) occurs during retinal degeneration in both the S334ter and P23H RHO rat models [11,17,18]. In the S334ter transgenic retina we have also determined that activation of the UPR is associated with increased expression of the JNK, Bik, Bim, Bid, Noxa, and Puma genes and cleavage of caspase-12 that, together with activated calpains, presumably compromise the integrity of the mitochondrial MPTP in ADRP photoreceptors [18]. In P23H-3 *RHO* rats there have been no cellular signaling pathways identified as being involved in the mechanism underlying photoreceptor degeneration with the exception of the UPR [11,17,19]. Therefore, there is a critical need to describe any pathways that are modified concomitantly with the activated UPR in P23H-3 *RHO* photoreceptors in order to validate new therapeutic targets.

The UPR is initiated by the activation of three signaling pathways (PERK, ATF6 and IRE1) and is required for controlling ER protein folding capacity and reestablishing homeostasis in the cell. Upon acute or unresolved ER stress, the UPR also triggers apoptosis, which eliminates any cell that is incapable of restoring proper protein folding and orchestrating a coordinated adaptive downstream response. Previously, we demonstrated that P23H-3 RHO photoreceptors have an activated ER stress-induced caspase-7 [17] and a consistent increase in Bip and CHOP gene expression [11]. These results suggested that ER stress persists in P23H-3 RHO photoreceptors, which might stimulate apoptotic signaling in these animals. Subsequently the persistence of the UPR has been confirmed in our other study [11]. However, apart from the UPR, other signaling pathways have been found to originate from the ER and those pathways have not been properly investigated. For example, the Ca^2+^-mediated signaling pathway could be triggered by the ER stress response leading to activation of calpains and caspase-12 [20,21]. The Bcl2 family proteins are known to be upregulated during UPR activation via the transcriptional activity of ATF4 and CHOP (Puma, Noxa and Bim). They can also be upregulated by activated JNK, which phosphorylates the BCL-2/BCL-XL proteins and additionally, promotes autophagosome formation by releasing the active beclin-1/PI3K complex from the ER. This complex is known to regulate ATG12–ATG5 formation and to promote the LC3-II conversion during the formation of autophagosomes [22]. Therefore, autophagy, the major degradation pathway after UPR activation in neuronal cells, could also be induced by ER stress. Both the PERK/eIF2α and IRE1 arms of the UPR have been implicated in the regulation of autophagy [23].

mTOR/AKT signaling is another example of a pathway that is tightly regulated by the UPR. We have previously reported that a T17M *RHO* retina that experiences UPR activation also demonstrates an elevation of mTor protein and a decrease in phosphorylated AKT [21]. It has been demonstrated that the UPR can activate mTOR *via* ATF6a signaling [24]. The ATF6 UPR pathway was found to be upregulated in both P23H and T17M RHO retinas [17,21], thus implying that similar to T17M RHO, P23H RHO photoreceptors could also have modified mTOR/AKT signaling.

Thus, several signaling pathways have been identified that are mediated by the activation of the UPR, which is found in P23H-3 *RHO* photoreceptors [11,17,19]. They could be modified either by ER stress or independently altered to contribute to the mechanism of ADRP. In both scenarios, these signaling pathways could provide alternative therapeutic strategies in the P23H RHO retina in addition to gene therapy against mutant rhodopsin. Therefore, the major focus of this study is to identify whether UPR activation in P23H RHO photoreceptors is accompanied by changes in Ca^2+^-induced signaling, autophagy, and mTOR/AKT pathways during ADRP progression and whether alterations in mTOR cellular signaling could be responsible for slowing the rate of retinal degeneration.

## 2. Materials and methods

### 2.1. Animal use

All animal procedures conformed to the Association for Research in Vision and Ophthalmology Statement for the Use of Animals in Ophthalmic and Vision Research, and were approved by the Institutional Animal Research Committee of the University of Alabama at Birmingham. The P23H-3 *RHO* (line 3) rats were kindly provided by Dr. LaVail (UCSF). Rats were housed in specific pathogen-free (SPF) conditions with a 12-hour light and 12-hour dark cycle. Animals were sacrificed by thoracotomy, and retinas were rapidly excised (removal of the lens), placed on an ice-cold plate, and stored at −80 °C.

### 2.2. RNA preparation and quantitative real-time PCR

Total RNA was isolated from individual SD and P23H-3 RHO (N = 4) retinas using an RNeasy Mini kit (Qiagen, Valencia, CA). We then used a high capacity cDNA reverse transcription kit (Applied Biosystems, Carlsbad, California, USA). Two cDNA reactions were prepared from each RNA sample and 10 ng cDNA was used for qRT-PCR using Applied Biosystems TaqMan assays that were validated for each selected gene on a One Step Plus instrument (Applied Biosystems, Foster City, CA). To analyze the samples, we compared the number of cycles (Ct) needed to reach the midpoint of the linear phase and normalized all observations to the GAPDH housekeeping gene. The replicated RQ (Relative Quantity) values for each biological sample were averaged.

### 2.3. Retinal protein extract for western blot analysis

We obtained retinal protein extracts from dissected retinas that were sonicated in a buffer containing 25 mM sucrose, 100 mM Tris–HCl, pH = 7.8, and a mixture of protease inhibitors (PMSF, TLCK, aprotinin, leupeptin, and pepstatin). The total protein concentration from individual retinas was measured using a Bio-Rad protein assay, and 40 μg of total protein was used to detect individual proteins. The detection of proteins was performed using an infrared secondary antibody and an Odyssey infrared imager (Li-Cor, Inc.). Antibodies against PUMA (7467S), ATG5 (8540P), ATG7 (78558P), LC3 (#2775), mTOR (2983P), caspase-3 (9662P), caspase-9 (9507S) and caspase7 (9492P) were purchased from Cell Signaling (1:1000). Antibodies against AIF (sc5586), Cytochrome C (sc13156), NOXA (sc11718), and active BAX, which detects only the active form of the BAX protein (sc23959) were purchased from Santa-Cruz Biotechnology (1:1000). Anti-caspase-12 (ab62484) and anti-pAKT (ab66138), which detects phosphorylated AKT, were purchased from Abcam (1:1000). We used an anti-BAX antibody (B8429) and an anti-Lamp2 (L0668) antibody from Sigma-Aldrich (1:1000). β-Actin was used as an internal control and was detected using an anti-β-actin antibody (Sigma-Aldrich).

### 2.4. Intraperitoneal injection of rapamycin and ERG analysis

A 10 mg/kg dose of rapamycin was used for injections. A water solution of 5% ethanol, 5% tween-20 and 5% polyethylene glycol 400 was used to dissolve rapamycin and was also used in the vehicle-treated control injections. Starting at P15, daily intraperitoneal injections (IPs) were performed in rats over the course of 10 days, during which no signs of weight loss were recorded. The ElectroRetinoGrams (ERG) of treated and untreated animals were recorded as previously described [25] at P30 and P45, which correlate with 2 and 4 weeks post-treatment, respectively. Briefly, rats were dark-adapted overnight, then anesthetized with ketamine (100 mg/kg) and xylazine (10 mg/kg). Their pupils were dilated in dim red light with 2.5% phenylephrine hydrochloride ophthalmic solution (Akorn, Inc.). The KC UTAS-3000 Diagnostic System (Gaithersburg, MD) was used to perform scotopic ERGs. The measurements were conducted using a wire contacting the corneal surface with 2.5% hypromellose ophthalmic demulcent solution (Akorn, Inc.). The ERG was performed at different intensities including 0 dB (2.5 cd*s/m2), 5 dB (7.91 cd*s/m2), 10 dB (25 cd*s/m2), and 15 dB (79.1 cd*s/m2).

### 2.5. Calpain activity assay

The detection of calpain activity was performed using the Calpain Activity Assay kit from BioVision in accordance with the manufacturer’s recommendations. The detection of the cleaved substrate Ac-LLY-AFC was performed using a fluorometer that was equipped with a 400-nm excitation filter and 505-nm emission filter.

### 2.6. Isolation of mitochondria from rat retinas

We separated the cytosolic fraction from the mitochondrial fractions from five individual P23H RHO and SD rats using the Mitochondria Isolation kit for Tissues (Thermo Scientific). The mitochondria were separated from the cytoplasm using the Dounce stroke method as recommended by the manufacturer. The protein concentration for each fraction was determined using a Bio-Rad protein assay. To confirm the absence of mitochondrial contamination in the cytoplasmic fractions, western blot was done using a COXIV antibody (Abcam). To confirm the absence of cytosolic contamination in the mitochondrial fractions, western blotting with an anti-actin antibody was employed (Sigma-Aldrich).

## 3. Results

We previously described the activation of the ER stress response in P23H-3 *RHO* rat retinas at P30 [17]. For this reason, the modulation of the other cellular signaling pathways in the P23H *RHO* photoreceptor was primarily studied at P30 and P40.

### 3.1. The hallmarks of Ca^2+^-induced signaling are upregulated in deteriorating P23H-3 RHO photoreceptors

**Fig. 1:**
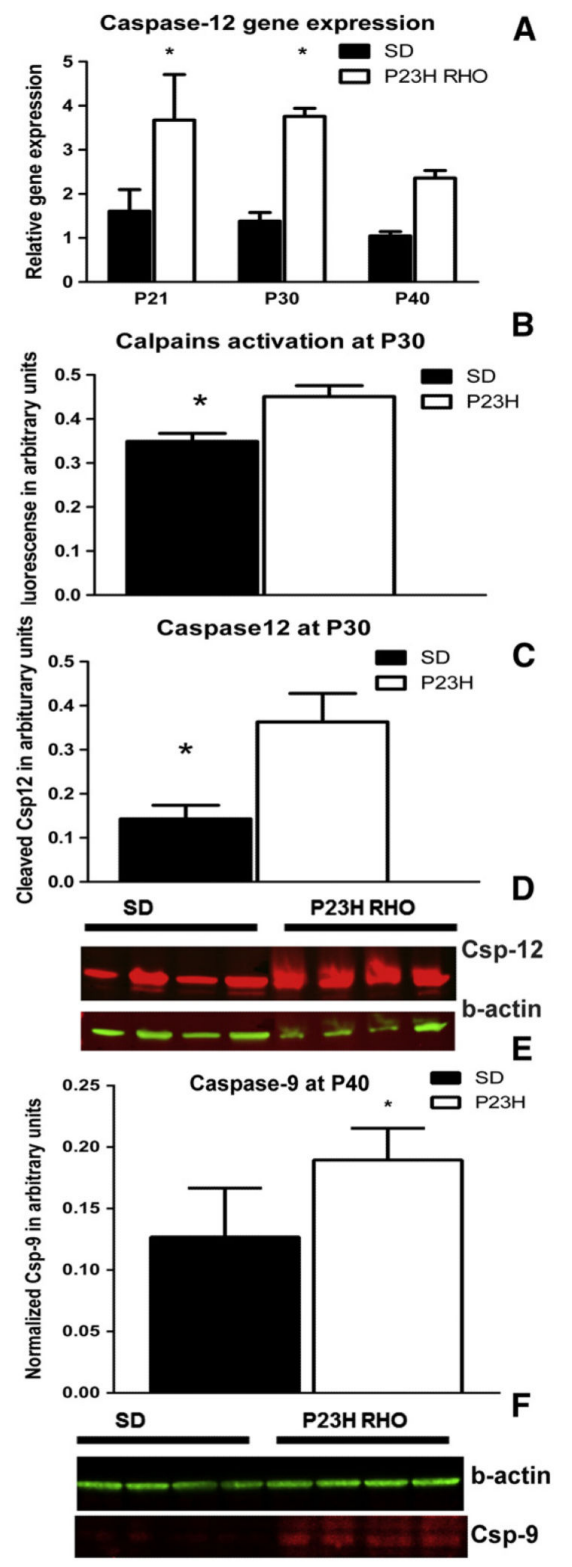
The activation of calpains, ER stress-induced caspase-12 and apoptotic protease activating factor-1-mediated caspase-9. (A) Relative caspase-12 gene expression in progressive P23H *RHO* retina. We analyzed caspase-12 gene expression by two-way ANOVA and found that at P21, P30 and P40 there was a greater-than 2-fold overexpression of caspase-12 mRNA in transgenic retinas (P < 0.05), N = 4. (B) We used the calpain μ and m activity assay, analyzed the results by a non-parametric *t*-test and found that the amount of the cleaved calpain substrate, Ac-LLY-AFC, was increased by 32% in transgenic retinas, N = 4. (C) The amount of cleaved caspase-12 was also elevated by 157% in P23H *RHO* retinas. (D) Images of western blots probed with anti-caspase-12 and anti-β-actin antibodies, N = 4. (A–C) These data suggest that calpain and caspase-12 are activated in the P23H *RHO* retina at P30 and point toward excessive cytosolic Ca^2+^ content that could perhaps result from persistent ER stress. (E) Detection of cleaved-9 in P40 transgenic retinas. We analyzed retinal protein extracts at P40 and found that the level of cleaved-9 was elevated in P23H *RHO* retina. The cleavage of caspase-9 was 40% higher in transgenic retina compared to SD (P < 0.05) and was coincided with the release of cytochrome *C* and AIF from the P23H RHO mitochondria. (F) Images of western blots probed with anti-caspase-9 and anti-β-actin antibodies, N = 4.

We investigated the expression of Calpain 1 and 2, as well as Caspase-12, and found no differences in calpain 1 or 2 mRNA compared with controls. However, we did observe significant differences in the caspase-12 gene expression at P21, P30 and P40 (Fig. 1A). At each time point, caspase-12 mRNA was induced by more than 2-fold compared to SD. At P21, the relative caspase-12 expression was 3.67 ± 1.03 in P23H *RHO* *vs.* 1.60 ± 0.49 in arbitrary units (a.u.) in SD, P < 0.05. At P30 it was 3.76 ± 0.18 in P23H RHO *vs.* 1.38 ± 0.19 a.u. in SD, P < 0.05 and at P40 it was 2.36 ± 0.17 in P23H *RHO* *vs.* 1.04 ± 0.09 a.u. in SD, P < 0.05.

An analysis of activated calpain 1 and 2 demonstrated that at P30, the activation of calpains was 30% higher in P23H *RHO* retinas compared to SD (0.45 ± 0.02 in P23H *RHO* photoreceptors *vs.* 0.34 ± 0.02 in SD a.u., P = 0.01) (Fig. 1B). Cleavage of caspase-12 in P23H *RHO* retinas was 2.6-fold higher compared to SD (0.36 ± 0.06 in P23H photoreceptors *vs.* 0.14 ± 0.03 in SD a.u., P = 0.02) (Fig. 1C).

### 3.2. The expression of Bcl-2 family proteins is modulated in P23H-3 RHO retina

**Fig. 2:**
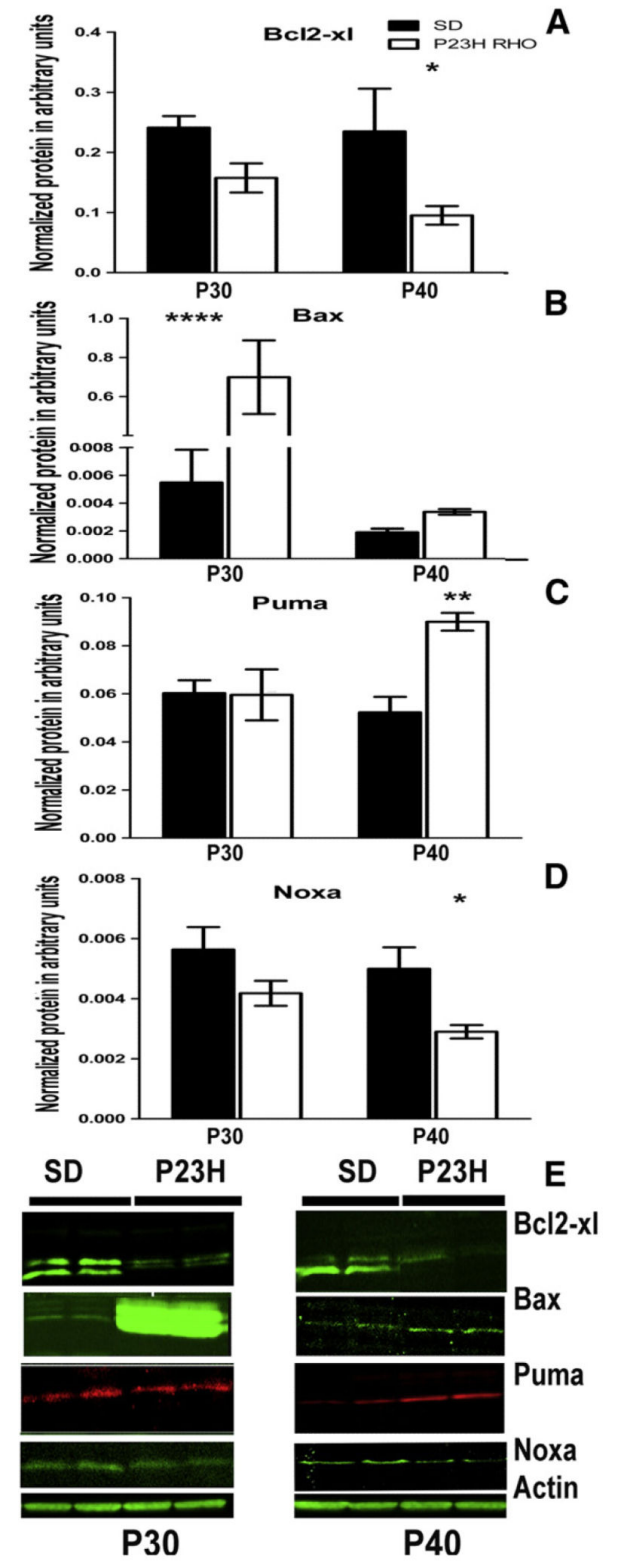
Changes in the expression of Bcl2 family proteins in the progressive ADRP retina. (A) We analyzed retinal protein extracts from SD and P23H *RHO* rats by two-way ANOVA and found that the BCL2-XL protein was downregulated at P30 and P40 in P23H *RHO* photoreceptors. At P40, we observed a 59% reduction in the amount of BCL2-XL protein (P < 0.05), N = 4. (B) BAX protein was significantly elevated by 1270% (P < 0.0001) at P30 and by 73% at P30 in the P23H *RHO* retina, N = 4. (C, D) PUMA and NOXA proteins had a unique pattern of expression. (C) The PUMA protein was elevated by 73% at P40 (P < 0.01) and (D) NOXA was diminished by 41% at P40 (P < 0.05), N = 4. (E) Images of western blots probed with ant-Bcl2-xl, BAX, PUMA, NOXA and β-actin antibodies, N = 4.

We analyzed Bcl-2 family protein expression by western blot analysis (Fig. 2) and found that the level of anti-apoptotic BCL2-XL protein was decreased in transgenic retinas by 0.6-fold (0.15 ± 0.02 in P23H RHO photoreceptors *vs.* 0.24 ± 0.003 in SD a.u.) at P30 and by 0.4-fold (0.095 ± 0.01 in P23H RHO photoreceptors *vs.* 0.23 ± 0.07 in SD a.u., P < 0.05) at P40. Alternatively, the expression of proapoptotic BH3-only proteins, such as BAX and PUMA, increased over time. The level of BAX was dramatically increased (136-fold) at P30 (0.68 ± 0.18 in P23H photoreceptors *vs.* 0.005 ± 0.002 in SD a.u., P < 0.0001). At P40, BAX protein levels were significantly decreased (0.003 ± 0.0002 in P23H *RHO* photoreceptors *vs.* 0.002 ± 0.0002 in SD a.u.). PUMA protein was not statistically different compared to SD at P30 (0.059 ± 0.01 in P23H RHO photoreceptors *vs.* 0.060 ± 0.005 in SD a.u.), but at P40, its expression was significantly elevated by 1.7-fold (0.09 ± 0.004 in P23H photoreceptors *vs.* 0.05 ± 0.006 in SD a.u., P < 0.01). The level of NOXA protein decreased over time. However, this decrease was not statistically significant compared to SD at P30 (0.004 ± 0.0004 in P23H RHO photoreceptors *vs.* 0.005 ± 0.0007 in SD a.u.). At P40, the level of Noxa was reduced by 40% (0.003 ± 0.0002 in P23H photoreceptors *vs.* 0.005 ± 0.0007 in SD a.u., P < 0.05).

### 3.3. Initiation of mitochondria-induced apoptosis in P23H RHO photoreceptors

**Fig. 3:**
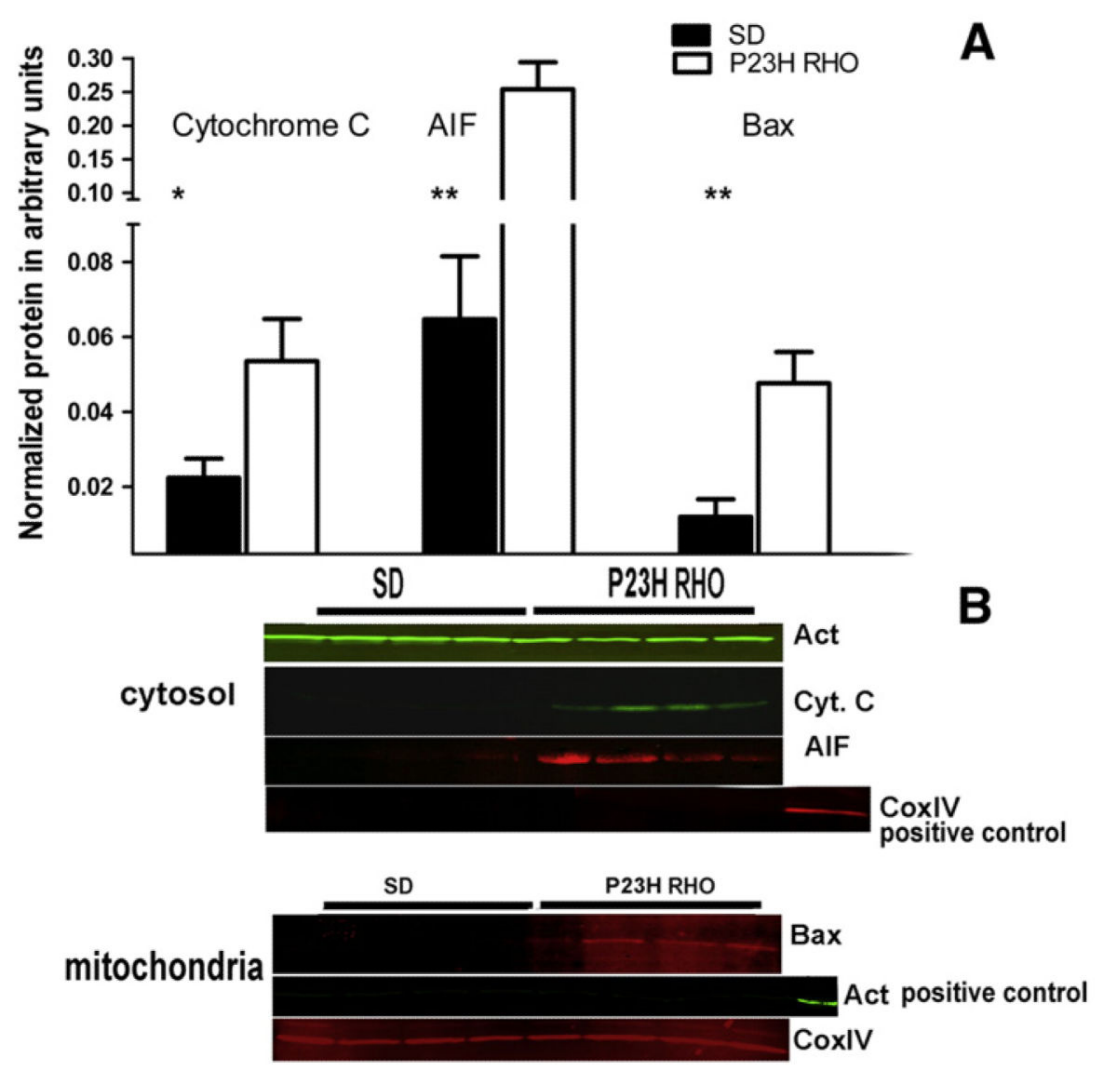
Mitochondria-induced apoptosis in the P23H *RHO* retina. We separated the retinal cytosolic and mitochondrial fractions (N = 4) and analyzed the cytosolic fraction by probing western blots with antibodies against cytochrome *C* and cleaved AIF. (A) Using a non-parametric *t*-test, we found that at P21 there was a release of cytochrome *C* from the P23H *RHO* mitochondria to the cytoplasm. The observed density was 140% higher in transgenic cytosol samples compared to SD (P = 0.02). In addition, we observed a release of cleaved AIF from P23H *RHO* mitochondria The observed density for the corresponding band was 290% higher in P23H *RHO* rats compared to SD, (P = 0.002). At P40, we detected the translocation of BAX protein into the mitochondria of P23H *RHO* photoreceptors. The density for the corresponding band was 327% higher in transgenic photoreceptors than in SD (P = 0.009). The observed time point for this translocation (P40) does not exclude the possibility that BAX protein translocates to the mitochondria at earlier time points. (B) Western blot images for blots treated with anti-cytochrome *C*, AIF and BAX antibodies experiment with running anti-β-actin control for the mitochondrial fraction (two last lanes are positive controls) and COXIV control for the cytoplasmic fraction (two last gels are positive controls).

We separated the mitochondrial fraction of photoreceptor cells from the cytosol in order to analyze mitochondrial cytochrome *C* release and the translocation of AIF into the cytosol (Fig. 3). We found that as early as P21 there was a greater-than 2-fold accumulation of cytochrome C in the cytosol of P23H RHO retina photoreceptors (0.053 ± 0.011 *vs.* 0.022 ± 0.005 in SD in a.u., P = 0.02). We also found the release of the AIF protein from P23H RHO mitochondria. The AIF content of the P23H cytosol was more than 4-fold higher than in SD (0.25 ± 0.04 in P23H RHO photoreceptors *vs.* 0.022 ± 0.02 in SD in a.u., P = 0.002). The cytochrome C and the AIF1 releases from the mitochondria were in agreement with elevation of the cleaved caspase-9 by 40% (0.19 ± 0.01 in P23H RHO photoreceptors *vs.* 0.12 ± 0.01 in SD in a.u., P = 0.03) at P40, Fig. 1E, F.

We found that BAX translocated to the mitochondria in P23H *RHO* photoreceptors at P40 and that the level of oligomerized BAX protein in the mitochondria of transgenic rats was 4.7-fold higher than in SD (0.047 ± 0.008 *vs.* 0.011 ± 0.005 in a.u., P = 0.009).

### 3.4. Autophagy is modified in the ADRP retina

**Fig. 4:**
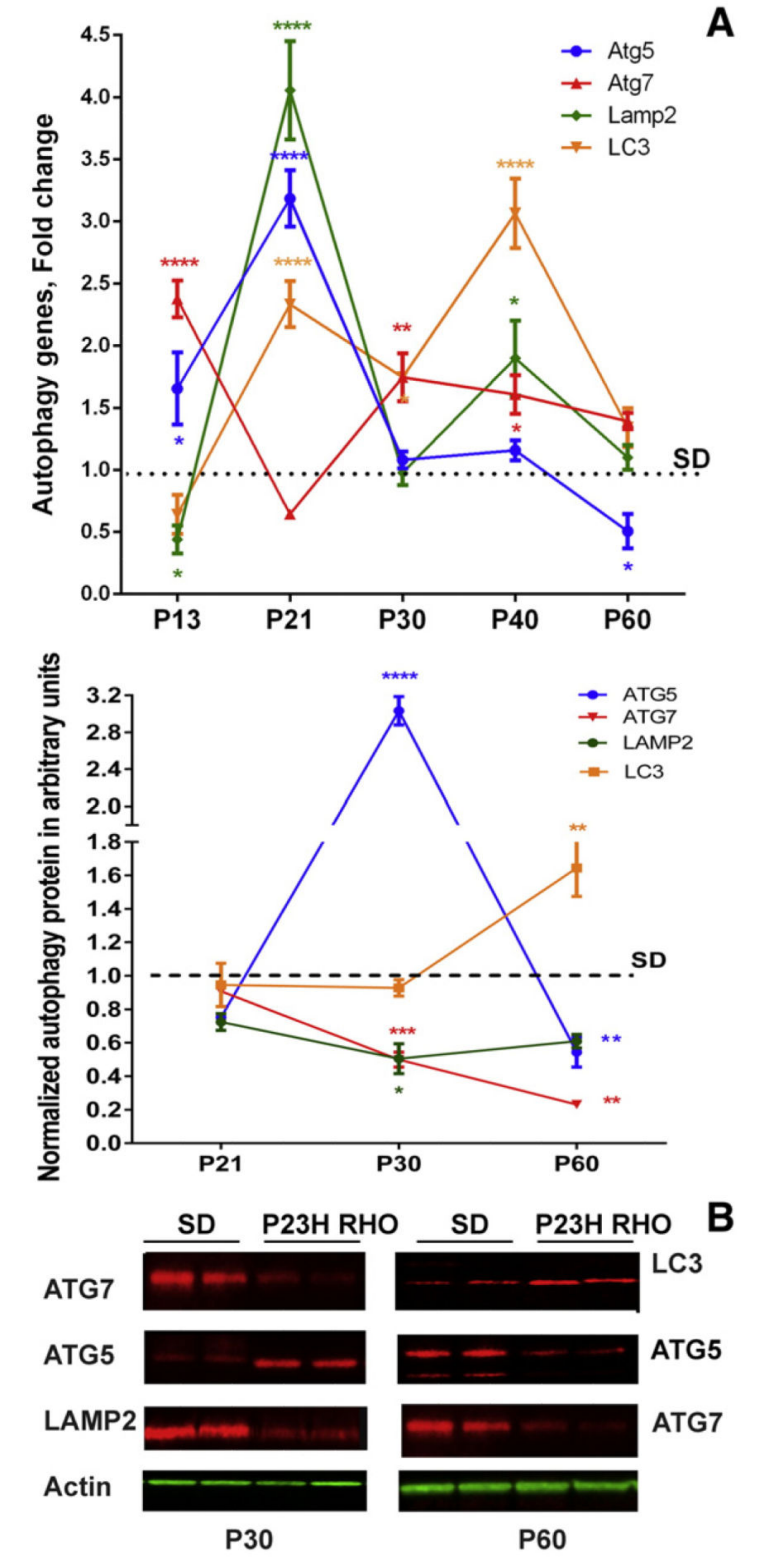
Autophagy gene and protein expression are modified in P23H-3 RHO photoreceptors in a multiphasic manner. (A) Atg5, Atg7, Lamp2 and Lc3 gene expression in P13, P21, P30, P40 and P60 P23H RHO retinas (N = 4). At P13, expression of Atg5 and Atg7 was upregulated in ADRP photoreceptors by 1.6 and 2.4-fold respectively, while expression of Lamp2 was downregulated by 2-fold. At P21, expression of all genes, except Atg7, was upregulated by 3-, 4- and 2.3-fold for Atg5, Lamp2 and Lc3, respectively. At P30, expression of ATG7 was the only one significantly higher compared with controls. At P40, Atg7, Lamp2 and Lc3 gene expression was significantly elevated by 1.6-, 1.9- and 3.0-fold. At P60, expression of all genes was similar to SD with the exception of Atg5, which was downregulated by 2-fold. (B) The level of ATG5, ATG7, LAMP2 and LC3 proteins was detected in P21, P30, P40 and P60 P23H-3 RHO retinas (N = 4) and the pattern of protein expression was found to be different from that of mRNA. At P21, no difference in ATG5, ATG7, LC3 and LAMP2 protein was observed in ADRP photoreceptors. At P30, we found that ATG5 protein levels were increased by 3.1-fold while levels of ATG7 and LAMP2 protein were decreased by 40% and by 50%, respectively. At P60, the level of LC3 protein was 60% higher in ADRP photoreceptors *vs*. SD photoreceptors, which was significant.

The SD and P23H-3 RHO RNA extracts from P13, P21, P30, P40 and P60 retinas were analyzed to detect expression of the Atg5, Atg7, Lamp2 and Lc3 autophagy genes (Fig. 4A). The results of this experiment demonstrated that the pattern of autophagy gene expression was not uniform. For example, in P13 retinas expression of Atg7 and Atg5 genes was increased by 2.3 ± 0.14 and 1.6 ± 0.29 fold, (P < 0.0001 and P < 0.5), respectively while expression of the Lamp2 was only 0.43 ± 0.11, P < 0.05 of SD. At P21, expression of Atg5, LC3 and Lamp2 was significantly increased and was 3.18 ± 0.22, P < 0.0001, 2.33 ± 0.18, P < 0.0001 and 4.05 ± 0.39, P < 0.0001, respectively compared to SD, however, expression of Atg7 did not differ significantly from SD retinas. At P30, Atg7 and Lc3 gene expression remained elevated by 1.74 ± 0.19, P < 0.01 and 1.75 ± 0.19, P < 0.01, respectively while expression of the Lamp2 and Atg5 genes was downregulated. At P40 expression of all genes, except Atg5, was significantly upregulated and was 1.6 ± 0.15, P < 0.05; 1.90 ± 0.30, P < 0.05 and 3.06 ± 0.27, P < 0.0001 fold higher than SD for Atg7, Lamp2 and Lc3, respectively. By P60 expression of all genes was diminished, however only the decrease in Atg5 gene expression was significant and was 0.5 ± 0.13 of SD P < 0.05.

We tested protein extracts from SD and P23H RHO retinas and found that the changes in the protein level of ATG5, ATG7, LAMP2 and LC3 did not correlate with alterations in the autophagy gene expression over the time (Fig. 4B). For example, all proteins with the exception of ATG5 at P30 and LC3 at P60 were either not significantly changed or significantly downregulated compared to SD. ATG5 was elevated by more than 3-fold (0.28 ± 0.023 in SD *vs.* 1.01 ± 0.078 in P23H-3 RHO, in a.u., P < 0.0001 at P30) and was downregulated by nearly 2-fold (0.45 ± 0.029 in SD *vs.* 0.247 ± 0.040 in P23H-3 RHO a.u., P < 0.01) at P60. ATG7 was significantly diminished at both the P30 and P60 time points and was 0.45 ± 0.074 in SD *vs.* 0.22 ± 0.044 in P23H-3 RHO a.u., P < 0.001 and 0.25 ± 0.015 in SD *vs.* 0.058 ± 0.0128 a.u. in P23H-3 RHO, P < 0.01, respectively. LAMP2 was also downregulated at P30 by over 50% and was 1.17 ± 0.17 in SD *vs.* 0.59 ± 0.093 in P23H-3 RHO a.u., P < 0.05. In contrast, the LC3 protein showed an increase of 64% at P60 and was 1.88 ± 0.21 in SD *vs.* 3.03 ± 0.27 in P23H-3 RHO in a.u., P < 0.01.

### 3.5. mTOR/AKT signaling is modified in progressive P23H RHO photoreceptors

**Fig. 5:**
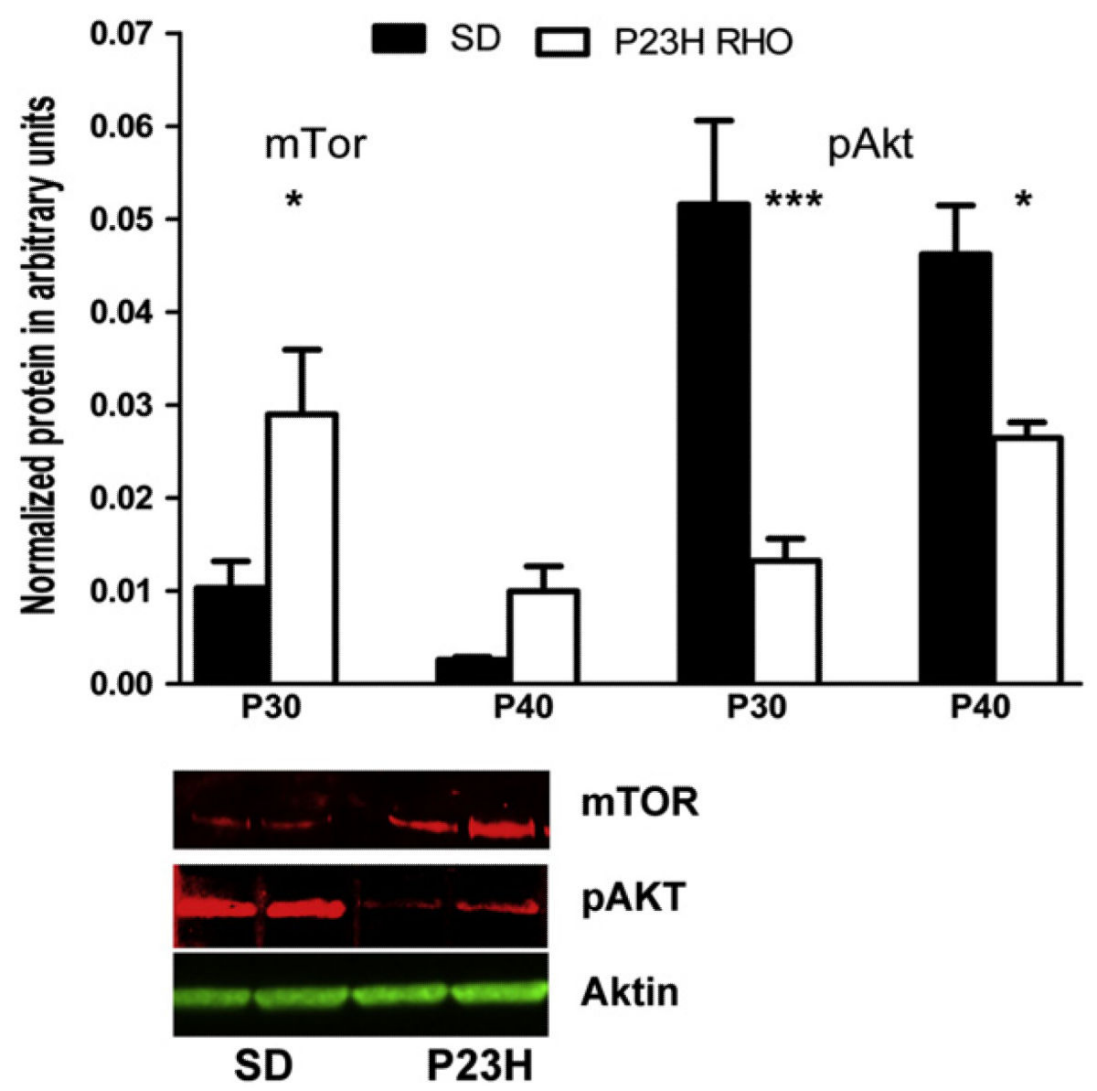
The mTOR/AKT pathway is modified in the progressive ADRP retina. We analyzed retinal protein extracts from SD and P23H *RHO* rats by two-way ANOVA and determined that mTOR protein expression was significantly elevated in the P23H *RHO* retina at P30 (by 290%; P < 0.05), N = 4. The level of pAKT was reduced at P30 by 75% (P < 0.001) and at P40 by 44% (P < 0.05), N = 4. Bottom: Images of western blots for P23H-3 RHO and SD at P30 probed with anti-mTOR, pAKT and β-Actin antibodies.

We analyzed mTOR and phosphorylated AKT (pAKT) in P30 and P40 retinas and found that the level of mTOR protein was elevated in P23H RHO animals by 290% at P30 (0.029 ± 0.002 *vs.* 0.01 ± 0.009 in SD a.u.), P < 0.05 and by 430% at P40 (0.009 ± 0.003 *vs.* 0.002 ± 0.0003 in SD a.u.) (Fig. **5**). In contrast, pAKT protein was significantly downregulated by 75% at P30 (0.013 ± 0.002 in P23H photoreceptors *vs.* 0.051 ± 0.009 in SD a.u.), P < 0.001 and by 44% at P40 (0.026 ± 0.04 in P23H photoreceptors *vs.* 0.046 ± 0.005 in SD a.u.), P < 0.05.

### 3.6. Injection of rapamycin decreases the level of mTOR and slows the rate of retinal degeneration

**Fig. 6:**
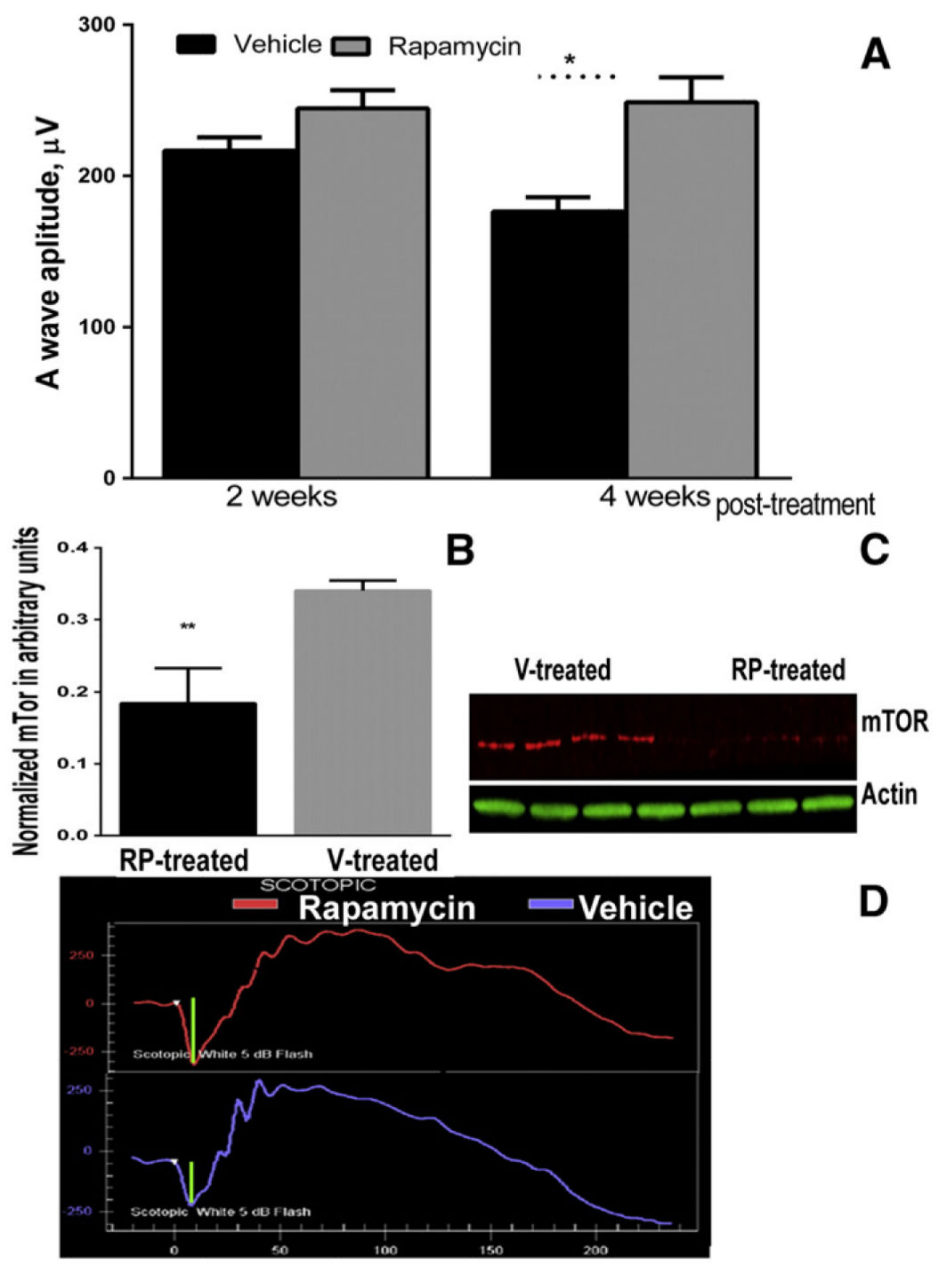
Injection with rapamycin slows the decline of scotopic a-wave ERG amplitude. (A) A 40% increased in a-wave amplitude of scotopic ERGs was observed in P23H-3 RHO rats IP-injected with rapamycin (RP) (N = 8) *vs*. vehicle (V)-injected (N = 6) rats, P < 0.05. There was no difference between a-wave amplitudes in RP-treated animals at 2 and 4 weeks while V-treated rats demonstrated a continued decline in a-wave amplitude. The B-wave amplitude in rats treated with rapamycin was not changed (not shown). (B). IP-injected rapamycin led to a decrease in mTOR levels in the P23H-3 RHO retina after 2 weeks post-treatment. Nearly a 2-fold decrease in the level of mTor was observed in RP-treated animals, P = 0.012. (C) Images of blots probed with anti-mTor and β-actin antibodies.(D) Images of the scotopic ERG amplitudes registered at 0 dB or 2.5 cd*s/m^2^in RP- and V-treated P23H RHO rats.

Knowing that rapamycin could modify mTOR signaling, we performed IP injections in P23H-3 RHO rats. Fig. 6 demonstrates results of the scotopic ERG analysis in RP-treated and V-treated rats. Analysis of scotopic ERGs revealed that a-wave amplitude was slightly increased in the RP-treated P23H-3*RHO* rats at 2 weeks post-treatment (216 ± 8.8 in V-treated *vs.* 245 ± 11.98 in RP-treated retinas) and was increased by 40% in P23H-3*RHO* rats at 4 weeks post-treatment compared with controls (176.4 ± 9.4 in V-treated *vs.* 249 ± 16.5 in RP-treated), P < 0.05. The b-wave amplitude of the scotopic ERG and the a- and b-wave amplitudes of the photopic ERG did not change in the RP-treated P23H-3 *RHO* rats. Analysis of retinal protein extracts demonstrated that the mTOR protein was downregulated in RP-treated animals by nearly 2-fold (0.18 ± 0.02 in RP-treated rats *vs.* 0.34 ± 0.008 in V-treated animals, P = 0.012.)

## 4. Discussion

Apoptosis has been shown to be the common pathway of photo-receptor cell death in several animal models of inherited retinal degeneration [26]. For example, in rd mice carrying mutant rod cGMP phosphodiesterase, the photoreceptors were found to die by apoptosis. In S334ter and P23H *RHO* rats, strong activation of calpain and poly(ADP-ribose) polymerase (PARP), concomitant with calpastatin downregulation, increased oxidative DNA damage and accumulation of PAR polymers was strictly correlated with the temporal progression of retinal degeneration [27]. We have previously reported UPR activation in both mutant RHO transgenic rat models. Therefore, because the UPR might affect general signaling networks in photoreceptor cells, this study focused on some of the pathways that have been reported to be tightly controlled by the UPR.

Supporting the hypothesis of a persistent UPR in P23H-3 *RHO* photo-receptors, the induction of Ca^2+^ signaling was observed at P30. The observed activation of calpain and caspase-12 at P30 suggests that Ca^2+^ release from the persistent UPR may occur. Calpain activity is highly controlled *in vivo* by multiple mechanisms, including phosphorylation by PKA, which negatively regulates calpain activity, and an endogenous inhibitor, calpastatin [28]. Therefore, the activation of calpain in P23H-3 RHO photoreceptors implies either excessive cytosolic Ca^2+^ and/or downregulation of PKA. Neither of these phenomena has thus far been tested in the P23H *RHO* retina.

The level of Bcl2 family proteins was also altered during the activated UPR in P23H-3 *RHO* retina. For example, the anti-apoptotic BCL2-XL protein was reduced at P30 and P40, which is indicative of the induction of transcriptional CHOP during UPR activation, as has been previously shown in P23H-3 *RHO* retinas [17,19]. Elevated CHOP may negatively regulate BCL2-XL gene expression [29]. In addition, p53 could be upregulated and, together with TATA binding protein, negatively control Bcl-2 gene expression by binding to the Bcl2 promoter TATA sequence [30]. Alternatively, the BAX protein was highly upregulated at P30, which suggests that the BAX/BCL2-XL ratio is significantly increased in transgenic rats. This in turn could determine the vulnerability of P23H-3 *RHO* photoreceptors to apoptosis that was confirmed in our experiments by detection of the cleaved caspase-12 and -9. In addition, we found that the BAX protein translocated to the mitochondria at P40 leading to MPTP. The observed time point for translocation (P40) does not exclude the possibility that BAX translocated at earlier time points. In line with this hypothesis, we observed the release of cytochrome *C* and AIF from the mitochondria to the cytosol of P23H *RHO* photoreceptors at P21. It is thus possible that this translocation could occur much earlier.

Other BH3-only proteins such as PUMA and NOXA also demonstrated altered expression at P30 and P40. Surprisingly, their changes in P23H-3 RHO photoreceptors were distinct. If the PUMA protein was increased at P40, then NOXA was decreased. Both proteins are well-known p53-inducible proapoptotic members and could participate in cell signaling differently [31]. For example, in normal cells, the MPTP induced by PUMA, but not NOXA, is mediated in part by a Ca^2+^ release from the ER [31]. However, upon expression of an oncoprotein such as E1A, induction of MPTP by NOXA occurs in cells in an ER-independent manner [31]. This fact provides additional evidence that the ER stress-induced release of Ca^2+^ to the cytosol occurs in the P23H *RHO* retina.

Autophagy has been implicated in various diseases including cancer, neurodegenerative diseases, pathogen invasion, as well as muscle and liver disorders. Interestingly, autophagy has been shown to be both beneficial and harmful [32]. In photoreceptor cell death, autophagy participates in the initiation of apoptosis [33] *via* a mechanism that in part depends on the Fas receptor. Autophagy has been shown to occur in photoreceptors as part of the basal processing of rod outer segments [34], as well as in animal models of hereditary retinal degeneration [35]. In our study we found that expression of autophagy-associated mRNAs was upregulated in the P23H-3 *RHO* retina and demonstrated a dynamic expression profile with a major increase in mRNA expression at P21. However, the UPR has been previously shown to be activated at P30 in P23H-3 RHO retina, and the PERK signaling pathway could perhaps be activated even earlier, leading to an upregulation of autophagy-related genes [36]. Regardless, by P60 a drop in the expression of autophagy-related genes was observed. However, the ER stress-induced Bip and CHOP mRNAs are significantly higher in P23H-3 *RHO* retinas at P60 as compared to SD [11]. That would suggest that a cellular mechanism other than the UPR controls autophagy gene expression in P23H-3 RHO photoreceptors.

Interestingly, protein levels of the corresponding autophagy genes did not mimic the dynamic alterations seen in the gene expression of the P23H-3 *RHO* retina. Recently, it was demonstrated that only 40% of the variation in protein concentration could be explained by knowing mRNA levels [37]. The remaining 60% of the variation in protein concentration could be determined by the rates of production and degradation of the participating molecules including the stability of mRNA, the average half-life of which is ten times less than that of protein. The role of miRNAs and RNA-binding proteins as potential mechanisms for regulating protein abundance was recently highlighted [37] and the influence of miRNA deregulation on chaperone-mediated autophagy was also revealed [38]. In the P23H-3RHO retina we observed a significant increase in autophagy gene expression at P21 that did not result in elevated levels of autophagy proteins. Moreover, at P30 the downregulation of LAMP2 and ATG7 proteins was observed. The only exception was ATG5 that was elevated more than 3-fold compared to controls at P30. Perhaps, ATG5 translation was not as affected by posttranslational modifications compared to ATG7, LAMP2 and LCA3 proteins whose levels may have been inhibited by either RNA–protein binding or by protein–protein interactions. Therefore, the elevated ATG5 protein level at P30 is a consequence of an increase in its mRNA at P21. For example, it was recently proposed that β-catenin could negatively regulate autophagy by suppressing formation of the autophagosome and forming the β-catenin–LC3 complex [39]. Another example is p62 that binds directly to LC3 leading to degradation of p62- and LC3-positive bodies [40]. This could happen in addition to a well-known negative regulation of autophagy by mTor, Bcl2, Beclin1 and p53 [41]. In agreement with these findings we found significant upregulation of mTOR protein at P30.

mTOR/AKt is known to be a negative regulator of autophagy [42], and its activity could attenuate autophagy and initiate lysosomal reformation. Recently, it has been found that during photoreceptor apoptosis in retinal degeneration in rd mice, the AKT survival pathway is inactivated [43]. In our ADRP rat model, we also observed downregulation of pAKT, which suggests that the inactivation of this pathway might have contributed to photoreceptor cell death. Thus, mTOR is highly upregulated in P30 P23H RHO retinas, which is in agreement with the activation of ATF6 [17]. With respect to the UPR, these data suggest that P30 is a critical time point for P23H-3 *RHO* photoreceptors, at which the decision is made by the cell whether or not to prolong the UPR and to switch from pro-survival to pro-death UPR signaling. For this reason, we decided to modulate the mTOR protein using peritoneal injections of rapamycin. We found that the injections lead to an increase in the scotopic a-wave ERG response at 4 weeks after treatment. However, this treatment protected photoreceptors from further functional decline instead of rescuing vision in ADRP rats. It also needs to be mentioned that only the rod photoreceptor cells were sensitive to this treatment and that photopic ERG response, typical for cone photoreceptors was not altered. This would suggest that the major biochemical imbalance associated with an increase in mTOR occurs specifically in rod photoreceptors and that downregulation of mTOR benefits the ADRP rods expressing P23H RHO.

Surprisingly, we found that inhibition of mTOR in treated retinas did not lead to an increase in autophagy protein production and that the ATG5, ATG7, LAMP2 or LC3 proteins were not altered. This fact would suggest that while downregulation of mTOR is sufficient to protect the P23H-3 RHO photoreceptors from functional decline to some degree, proteins other than mTOR are also able to negatively regulate the level of autophagy in the P23H-3 *RHO* retina.

## 5. Conclusions

The current findings demonstrate that modulation of cellular networking occurs in P23H RHO photoreceptors concomitantly with the activation of the UPR. We demonstrated that during ADRP progression, mTOR/AKT and autophagy signaling, along with the expression of Bcl2-family proteins, was altered, and that these alterations coincided with changes in Ca^2+^-regulated calpain and caspase-12 activation. In addition, we found that BAX translocated to the mitochondria and that this appeared to lead to mitochondria-induced apoptosis during ADRP progression. These data indicate the potential link between activation of the UPR and modulation of cellular signaling networks in P23H-3 *RHO* photoreceptors. However, additional experiments, including the modulation of UPR signaling by activating or inhibiting individual UPR arms in P23H-3 *RHO* retinas are necessary to uncover their relationships in the progressive ADRP retina. These results also point to a potential therapeutic target for blocking ADRP progression in P23H-3 *RHO* rats. Using mTOR inhibitors such as rapamycin that have been successfully tested in mice [44-46], and now in rats, could be a promising therapeutic approach for treating ADRP.
